# Effect of regulated and controlled deficit irrigation on yield and yield response factor of processing tomato

**DOI:** 10.1186/s12870-025-08065-6

**Published:** 2026-01-22

**Authors:** M. A. Badr, Eman Ali, S. R. Salman

**Affiliations:** 1 Plant Nutrition Department, National Research Center, Cairo, Egypt; 2 Vegetable Research Department, National Research Center, Cairo, Egypt

**Keywords:** Water stress, Tomato yield, Water productivity, Yield response factor

## Abstract

Deficit irrigation (DI) can help conserve water and alleviate the issue of water scarcity. For this purpose, two field experiments were conducted in 2023 and 2024 to evaluate a strategy involving reduced water use without compromising the yield production of processing tomato (cv. Castle Rock) in a typical arid environment of Egypt. In 2023, regulated deficit irrigation (RDI) was applied at 100% (V100), 80% (V80), 60% (V60), 40% (V40), and 0% (V0) of evapotranspiration (ET) following plant establishment. In 2024, controlled deficit irrigation (CDI) was applied at 100% (V100) or 50% (V50) during the whole growing season, 50% reduction up to the first fruit set, then 100% restoration (V50-100), and 100% until the beginning of ripening, then 50% reduction (V100-50), and 0% (V0) as in the previous year. In 2023, RDI decreased yield by 9.1, 26.2, and 51.1% for treatments V80, V60, and V40, respectively, with a remarkable increase in water productivity (WP) for all treatments compared to V100. In 2024, CDI included a reduction of water early in the season (V50-100) did not lead to significant losses in yield but resulted in a water saving of 25% compared to (V100), while the yield was negatively affected by the reduction of water late in the season (V100-50). Water productivity was positively affected by both treatments but V50-100 appreciably increased WP. Yield response factor (*K*y) with values less than one in both years showed relative tolerance of tomato to water deficit. The sensitivity of tomato to deficit irrigation was higher when water was applied at different intensities with RDI (*K*y = 0.96) than at individual growth stages with CDI (*K*y = 0.87). These results indicate the priority of deficit irrigation during the vegetative growth stage to improve crop yield, conserve water and enhance water productivity.

**Supplementary Information:**

The online version contains supplementary material available at 10.1186/s12870-025-08065-6.

## Introduction

 The continuous escalation in the demand for freshwater on a global scale to meet the needs of natural ecosystems will force irrigation to operate under water scarcity. In many areas of the world, irrigation processes are still practiced until now with disregard to basic principles of resource preservation. The increasing competition for water resources between agriculture and other sectors compels the adoption of irrigation strategies in arid regions, which may allow saving irrigation water and still maintaining satisfactory levels of production [1]. Under such conditions, there is an urgent need for a water saving strategy that must be seen as the major threat to food security in the future. Soil and water management is likely the best option in most agricultural systems for increasing the efficiency of water use [2, 3].

The process of crop water use has two main components of water losses: evaporation from the soil and the plant, as well as all the losses resulting from the distribution of water to the land [4, 5]. However, reducing crop ET without a penalty in crop production is much more difficult because evaporation from crop canopies is tightly coupled with the assimilation of carbon [6, 7]. Thus, some of the water losses are unavoidable and are needed to be minimized with efficient irrigation methods and by appropriate management.

The main approach to achieve this goal involves adopting more efficient irrigation techniques, such as drip irrigation, which reduce evaporation losses [3], as well as using deficit irrigation strategy [8, 9]. In this way, the entire root zone is irrigated with lower amounts of water while the minor stress that develops has minimal effects on the yield. Regulated deficit irrigation is an alternative for areas where DI evidenced a negative effect on yield, as it seeks to optimize the use of water stress only in noncritical periods of the development of the plant. The timing and stage at which stress is imposed, as well as the intensity of stress also influence water productivity. This mode of irrigation requires close control of the timing and level of water deficit so that water deficits can be imposed at times when it has a minimum effect on yield [10]. In contrast, CDI is often considered more favorable than RDI, particularly under arid conditions, as it maintains a minimum water supply throughout the growing season, ensuring sustained physiological activity and more stable yields by avoiding severe stress at sensitive growth stages.

Tomato is one of the most widely cultivated vegetables globally, valued for its nutritional content and economic importance. However, water scarcity poses a significant challenge to tomato production, necessitating efficient irrigation strategies. Tomato plants have a relative ability to tolerate water stress, allowing for deficit irrigation management, as they can tolerate drought to some extent [11–13]. Many studies showed that the response of tomato yield to deficit irrigation at various growth stages was different, depending on the period and the degree of water deficit [14–16].

However, the tomato plant is particularly sensitive to water deficit during reproductive development, as it causes physiological disturbances leading to the loss of flower buds and open flowers, as well as a reduction in fruit set, which has a negative impact on commercial yield [14, 17]. Similarly, Jiang et al. [18] confirmed an acceptable balance between high water productivity and yield, supplying 2/3 of full irrigating at flowering and fruit development. It was reported that imposing a certain degree of water stress during the vegetative stage had no adverse effects on tomato yield and fruit quality [19, 20]. Moreover, tomato yields were highest when moderate water deficiency was applied at the flowering and fruiting stages, whereas water deficiency during the expansion to color change stages of processing tomatoes significantly reduced tomato yield. This suggests that tomatoes are less water-tolerant during the flowering and fruiting stages [21]. Regulated deficit irrigation may also maintain the size and weight of processed tomatoes and increase carotenoid levels [22].

Regulated deficit irrigation as a proven water-saving irrigation method, may be feasible for the water-saving production of processing tomatoes. RDI is a strategy for the control and utilization of water to improve WUE by reducing irrigation water during the period of crop fertility when the crop is not sensitive to water stress [23]. Few studies were reported about the application of DI in water yield relations of tomato, and so far as the literature reported, no modeling equations have been evaluated for the effects of different degrees of water deficit at various growth stages. The aim of the present study was to (i) determine the effect of deficit irrigation throughout the growing season and at different growth stages on yield and yield-water relationships of processing tomato (ii) develop an irrigation strategy that ensures water savings to irrigate more land, especially under desert agro-systems. This aspect is of utmost importance given the difficulty of providing more water needed for the agricultural sector especially in arid and semi-arid regions.

## Materials and methods

### Site and soil description

Field experiments were conducted on a vegetable farm located in the Serapium area, Ismailia province, east of the Nile Delta, Egypt, during the late summer growing season (August-December) 2023 and 2024 using a drip irrigation system. This area is a desert region included in the agricultural expansion program and has recently become productive land for many crops. The site is located in the arid climate at latitude 30°58 N and longitude 32°23 E with an elevation of 13 m above mean sea level. The area experiences a hot and dry climate with very little rainfall throughout the year. Temperatures can be very high in summer (June to August), often exceeding 35 °C during the day, with little relief at night. Winter months (November to February) are mild, with daytime temperatures ranging from 15 °C to 25 °C and cooler nights, sometimes dropping to around 10 °C or lower. The mean monthly evapotranspiration ranged from 6.9 to 2.5 mm in the respective cropping season. The climate parameters recorded from August to December during both growing seasons are summarized in (Table 1). The soil was loamy sandy in texture which was classified as an (*Entisol-Typic Torripsamments*), composed mainly of sand 84.5% sand, 11.2% silt and 5.3% clay. The average soil water content at field capacity from the surface soil layer down to 80 cm depth at 20 cm intervals was 0.18 (v/v), and the permanent wilting point for the corresponding depths was 0.08 (v/v), respectively. Average available N, P, and K from the surface soil layer down to 60 cm depth at 20 cm intervals were 12, 7, and 43 mg kg¹ soil, respectively, before the initiation of the experiment.

**Table 1:** Average monthly maximum (*T*_*max*_) and minimum (*T*_*min*_) temperature, relative humidity, rainfall, evapotranspiration (ET_0_), and wind speed during the growing season

Month	T_max_ (^o^C)	T_min_ (^o^C)	Relative humidity (%)	Rain fall (mm)	ET_0_ (mm day)	Wind speed (km h^− 1^)
2023						
August	36.1	22.0	53	0.0	6.7	15.6
September	33.5	20.2	50	0.1	5.9	16.1
October	30.5	17.4	56	2.0	4.6	15.2
November	26.4	13.5	59	5.5	3.2	15.6
December	21.6	9.7	60	7.4	2.5	15.9
2024						
August	35.3	21.9	54	0.0	6.9	15.6
September	33.2	19.6	53	0.0	6.4	15.8
October	32.7	17.7	57	2.3	4.6	15.7
November	26.4	14.4	59	7.7	3.4	15.9
December	21.9	9.7	60	2.9	2.6	15.8

### Experimental design and treatments

Processing tomato plants were subjected to five irrigation treatments based on crop evapotranspiration (ETc) in a complete randomized block design with three replicates. The drip tubing system “Evergreen Company, Cairo, Egypt” was used to irrigate the plants over two years (2023/2024). The treatments included regulated deficit irrigation (RDI) in 2023 and controlled deficit irrigation (CDI) in 2024 under the field conditions (Table 2). RDI involved a consistent reduction in water supply throughout the growing season, while CDI maintained a consistent reduction in water supply throughout definite growing stages.

**Table 2:** Description of irrigation treatments applied to tomato in two growing seasons

Year	Treatments	Description	Water applied (mm)
2023	V100	100% ETc (full irrigation)	374
	V80	80% ETc of full irrigation	314
	V60	60% ETc of full irrigation	240
	V40	40% ETc of full irrigation	165
	V0	No irrigation after plant establishment	47
2024	V100-100	100% ETc (full irrigation)	386
	V50-50	50% ETc during the whole season	206
	V50-100	50% ETc reduction up to 1st fruit set, 100% ETc restoration	303
	V100-50	100% ETc reduction up to start of maturity stage, 50% ETc restoration	318
	V0	No irrigation after plant establishment	55

Before tomato transplanting, drip tubing (twin-wall, 15 mm inner diameter, in-line drippers at 40 cm distance delivering 2.5 L h^–1^ at operating pressure 100 kPa) was laid out along each row at 100 cm apart in the center of the soil beds. Tomato seedlings (*Solanum lycopersicum* L.) of the cultivar “Castle Rock”, commercially provided by Korma Seed Co., Cairo, Egypt, were sown in early July in both years in seeding trays under greenhouse conditions. The seedlings were grown for 45 days and then directly transplanted to the main field at 25 cm intervals along drip lines (40000 plants ha^–1^) in all the treatments in the middle of August. Plants were arranged in north-south-oriented soil beds pre-furrowed to receive 40 t ha^–1^ of organic manure in plots (4.5 m wide × 15.0 m long). All treatments received the same amount of phosphorus, 150 kg P ha^–1^ as single super phosphate and 250 kg K ha^–1^ as potassium sulphate before transplanting, which was incorporated into the soil beds. Nitrogen fertilizer, 240 kg N ha^–1^ was applied as ammonium nitrate in water-soluble form at 7 day intervals in the drip irrigation system using a venturi-type injector. Fertigation events of N were started two weeks after planting in 12 equal doses and stopped 30 days prior to the end of the growing season.

### Estimation of crop water requirement

Meteorological data were calculated from the weather station of the Central Laboratory of Agricultural Climate for Ismailia province located near the experimental field. The irrigation water was schedule based on estimated the water requirement of the plants. The FAO Penman–Monteith semi-empirical formula (ETc = ET₀ × *K*c) as described by Allen et al. [24] was used to calculate the (ETc). The actual ETc was based on the product of ET_0_ and crop coefficient (*K*c) for different months based on crop growth stages using values for tomato (*K*c initial = 0.45, *K*c developmental = 0.75, *K*c middle = 1.15, *K*c maturity = 0.85) for growth stages 30/40/40/25 days [24]. The values of ETc were reduced by 40% during the intial stage of growth and by 20% during rest of the growing season to represent the percentage of the wetted area resulting from the drip line spacing in the field. The percentages of wetted area were determined as the average horizontal area wetted of the crop root zone as a percentage of the total crop area. Cumulative ETc for the different irrigation treatments and the relative quantity of water saving during the whole growing period were presented in (Table 2). In both years, the plants were irrigated daily during the initial stage of growth to encourage establishment, but thereafter irrigation frequency was running at 2–3 day intervals. The irrigation process was ceased at 120 DAP (15 days before the last picking) to prevent secondary growth.

### Yield response factor

The functional relationship between crop yield and water use is called the water-production function (ratio of actual to maximum ET) that limits the crop yield, assuming that all the other factors are at the optimum level. Seasonal values of the yield response factor (*K*y) were calculated for each experimental year using the equation proposed by (Stewart et al., 1977) [25] as follows:

1 $$\:1-\left(\frac{\mathrm{Y}\mathrm{a}}{\mathrm{Y}\mathrm{m}}\right)=Ky\:\left(1-\frac{\mathrm{E}\mathrm{T}\mathrm{a}}{\mathrm{E}\mathrm{T}\mathrm{m}}\right)$$

where Ym (kg ha^− 1^) and Ya (kg ha^− 1^) are maximum (that obtained from fully irrigated treatment) and actual yield, respectively, ETm (mm ha^− 1^) and ETa (mm ha^− 1^) are maximum (that obtained from fully irrigated treatment) and actual ET, respectively, *K*y is the yield response factor, that is defined as the decrease in yield per unit decrease in ET [25]. According to the *Ky* calculation, *K*ss was calculated by the Eq. (1), replacing Ym with maximum total dry biomass (SSm) and Ya with actual total dry biomass (SSa) as follows:

2 $$\:1-\left(\frac{\mathrm{S}\mathrm{S}\mathrm{a}}{\mathrm{S}\mathrm{S}\mathrm{m}}\right)=Kss\:\left(1-\frac{\mathrm{E}\mathrm{T}\mathrm{a}}{\mathrm{E}\mathrm{T}\mathrm{m}}\right)$$

where *K*ss indicates the biomass response factor, which is the correlation factor between relative total dry biomass loss and relative ET reduction.

### Measurements of crop parameters

Maximum biomass production was determined by harvesting three representative plant per treatment replicate at 90 DAT. Plant growth components were determined from 10 randomly selected plants in each plot, including total fresh fruit yield per plant, fruit number per plant, and average fruit weight per plant. At harvest, three representative selected plants were sampled from each experimental plot and plant parts (stems + leaves + fruits) were dried in a thermo-ventilated oven at 70 ◦C until constant weight, for dry biomass measurement. Harvesting of the tomatoes was made on the last week of November until the end of December for both seasons. Total fresh fruit yield was recorded on at least 50 plants in a row in each treatment in all the replications, and data were presented as tons per hectare.

### Water productivity

Water productivity is an indicator related to the water consumed by crops as to produce a certain yield and calculated for each irrigation treatment as the ratio between total fruit yield at harvest and total water used, as measured by water balance equation:

$$\:\mathrm{W}\mathrm{P}=\left(\frac{\mathrm{y}\mathrm{i}\mathrm{e}\mathrm{l}\mathrm{d}\:{\mathrm{k}\mathrm{g}\:\mathrm{h}\mathrm{a}}^{-1}}{\mathrm{W}\mathrm{a}\mathrm{t}\mathrm{e}\mathrm{r}\:\mathrm{a}\mathrm{p}\mathrm{p}\mathrm{l}\mathrm{i}\mathrm{e}\mathrm{d}\:\left(\mathrm{m}\mathrm{m}\right)}\right)$$

where WP is water productivity (kg ha^− 1^ mm^− 1^) ET is the seasonal plant evapotranspiration (mm).

### Statistical analysis

All data were subjected to the analysis of variance (ANOVA) appropriate to the experimental design to evaluate the effects of treatments on tomato yield, total dry biomass, shoot dry weight, N and P uptake. CoStat (Version 6.311, CoHort, USA, 1998–2005) [26] was used to conduct the analysis of variance. Comparison of treatment means was carried out using the least significant difference (LSD) at a 5% probability level. Regression analysis was performed between total seasonal water use and total fruit yield of the crop for both seasons.

## Results

### Climate trend and irrigation variables

Although most tomato plants grow and produce best in sunny summer weather, it is possible to grow tomatoes in late summer, particularly in most hot climates. During the growth season, the values of climate parameters differed from (August to December), where the greatest differences were recorded in Des, both for temperature and relative humidity. During the cropping cycle, one can point out sparse precipitation that occurred without effective amounts for both seasons. The air temperature was higher during the first part of the cycle, in August, when the max temperatures were almost over 35 °C. Afterwards, in September, the daily min and max temperatures decreased and followed the same trend till the end of December. The described climate parameter, though typical for that area, reduced crop water use due to the low values of daily ET_0_ from the planting till harvest and made a clear differentiation of the compared irrigated theses.

### Tomato yield and TDM

The yield components, including biomass production, fruit weight, harvest index, and WP were significantly affected (*P* < 0.05) by deficit irrigation treatments applied in 2023 (Table 3). Yield was severely depressed by soil water deficit when irrigation was stopped early in the season (V0) in both years, as the plants failed to develop under drier conditions. RDI resulted in a slight reduction in fruit yield by 9.1% in V80, while in V60 and V40 the yield was strongly reduced by 26.2% and 51.1% compared to V100 respectively. The accumulation of TDM also decreased with increasing deficit irrigation, but the differences compared to the V100 were much smaller and amounted to 7.4, 22.8, and 41.8% with V80, V60, and V40, respectively. Average fruit weight was also affected by deficit irrigation, leading to a noticeable decrease in the overall fruit size. This reduction not only impacts yield but also has potential repercussions on market value and consumer preference, especially at V60 and V40, which were significantly reduced compared to the other treatments. The relationship between fruit yield and TDM was very strong (R² = 0.98), indicating that the crop regulates its fruit yield proportional to total dry matter production.

**Table 3:** Total yield, biomass production, fruit weight, harvest index and WP of tomato as affected by deficit irrigation treatments in 2023 for RDI and 2024 for CDI

Irrigation treatments	Biomass (t ha^− 1^)			Fruit weigh (g fruit^− 1^)	Harvest index	WP kg ha^− 1^ mm^− 1^
	Fruit	Shoot DW	Total DW			
2023 (RDI)						
V100	58.80a	1.82a	4.76a	98a	0.62	157
V80	53.45b	1.74a	4.41b	92b	0.61	170
V60	43.38c	1.52b	3.69c	85c	0.59	181
V40	28.74d	1.19c	2.77d	67d	0.57	174
V0	5.59e	0.48d	0.78e	45e	0.39	119
2024 (CDI)						
V100	62.25a	1.93a	5.04a	97a	0.62	161
V50	45.76c	1.65b	3.94c	82c	0.58	222
V50-100	59.87a	1.90a	4.89a	95a	0.61	197
V100-50	51.64b	1.76b	4.34b	87b	0.58	165
V0	7.89d	0.67c	1.11d	43d	0.39	143

Values within the column followed by different letters are significantly different based on least significant difference (LSD) at *P* ≤ 0.05

In 2024, deficit irrigation at a reduced rate (50% ETc) during the whole growing season (V50) significantly reduced yield by 26.5% compared to V100, as many small green fruits were aborted, besides the decline in the average fruit weight. On the other hand, different degrees of yield reduction were observed in the treatments with deficit irrigation at one growth stage (early or late stage-CDI) compared to full irrigation. Early-stage CDI (V50–100) does not induce significant losses in fruit yield, while reduction of water during the late stage (V100–50) resulted in a significant 17.1% lower yield compared to V100.

Similarly, TDM showed significant differences between irrigation treatments, especially in prolonged deficit irrigation (V50) and in late reduction of water, which were 22.0% and 14.0%, respectively lower than in V100, while early reduction of water (V50-100) resulted in lower losses in final dry biomass (3.1%). Biomass allocation and total biomass index decreased significantly in response to (V50) and late-stage water reduction (V100-50), which was associated with reduced biomass distribution on fruits and hence lower yield. Mean fruit weight was not significantly affected by deficit irrigation except for V50 and V05-100, which decreased significantly compared to V100. The harvest index with V100-50 was almost close to V100, which may be associated with favorable growing conditions, while it decreased significantly with other treatments. Similar to the first season, the relationship between fruit yield and TDM was very strong (R² = 0.98), confirming the high correlation between both parameters.

### Water productivity and production function

The effects of different irrigation treatments on tomato yield in terms of WP varied widely in both years (Table 3). In 2023, RDI induced increases of WP values as much as 8.3, 15.0, and 10.8% for V80, V60, and V40, respectively, compared to V100 which resulted in the lowest value. Despite the yield reductions under all deficit irrigation levels, maximum value of WP corresponded to irrigation treatment (V60) indicating that deficit irrigation can still be a viable strategy to enhance water use efficiency. In 2024, maximum WP was found in the treatment with the minimum water supply (V50), but only late stage CDI (V100-50) did so without significant yield reduction compared to V100. Although the savings in water was almost equal in both V50-100 and V100-50, but WP was markedly higher by 22.3% versus 2.3% for both treatments, respectively, compared to V100.

**Fig. 1:**
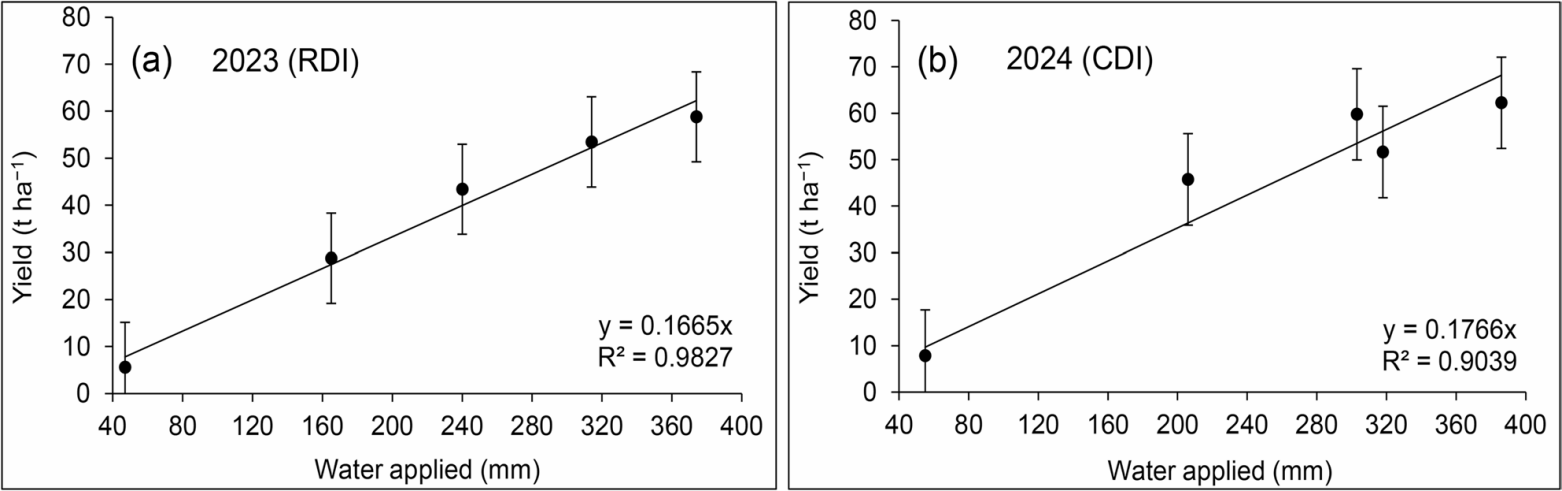
Relationship between total tomato yield and seasonal water applied in 2023 for RDI (**a**) and 2024 for CDI (**b**) through regression analysis

The relationship from regression analysis between seasonal ETc and fruit yield in both years was shown by a linear function (Fig. 1). As expected, the relationships between fruit yield and ETc were linear (a single line represented all five irrigation treatments applied in both years). The production function (total amount of irrigation water vs. fresh fruit yield) through linear regression analysis, showed a significant correlation coefficient (*R*^2^ = 0.98 and 0.90) for RDI and CDI, respectively. Over the range of water inputs, 47–55 mm for V0 to 374–386 mm for V100 between planting and harvest, tomato yield increased by about 167 and 177 kg ha^− 1^ for each mm of water applied for RDI and CDI, respectively.

### Yield response factor

The relationship between crop yield and water use was determined through an empirical model that links the relative yield decrease with the corresponding relative ET reduction, and the product of this model is a yield response factor (*K*y). This factor was calculated in this experiment for both total yield (*K*y) and total dry matter (*K*ss) produced by the crop for both RDI and CDI (Table 4). In 2023, the treatments with RDI showed persistent higher *K*y and *K*ss values with increasing levels of deficit irrigation, indicating that they were less tolerant under higher water stress. However, the treatment with V80 showed a minimum value compared to the maximum value with V40 or even greater with V0 when water supply was cut off very early in the season. In 2024, the treatments with CDI showed lower *K*y and *K*ss factors in V50–100 versus higher values for the same parameters in V100–50 which indicates to the sensitivity of tomato to deficit irrigation in the last part of the crop cycle.

**Table 4:** Yield response factor for yield *K*y and total dry biomass *K*ss of tomato for each individual treatment applied in 2023 for RDI and 2024 for CDI

Year	Treatments	Ky	Kss	Year	Treatments	Ky	Kss
2023	V100	--	--	2024	FI	--	--
	V80	0.56	0.44		V50	0.55	0.47
	V60	0.72	0.64		V50-100	0.18	0.14
	V40	0.91	0.75		V100-50	0.94	0.78
	V0	--	--		V0	--	--

**Fig. 2:**
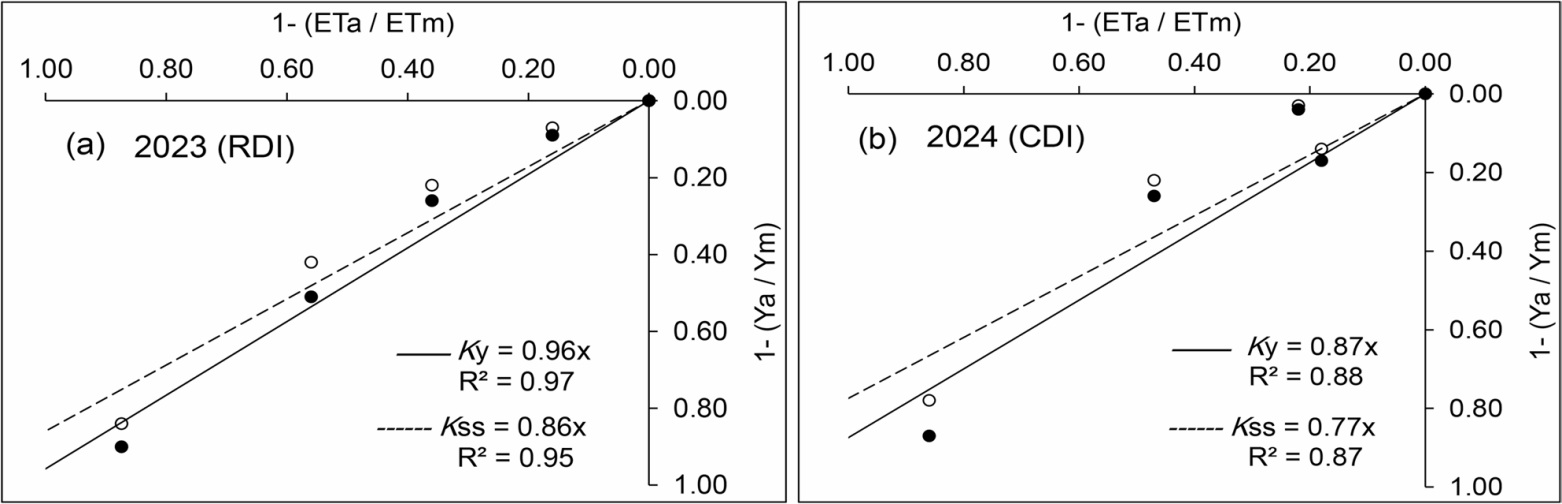
Relationship between relative yield decrease (solid curve) and total dry biomass decrease (dotted curve) as a function of relative ET decrease in 2023 for RDI (**a**) and in 2024 for CDI (**b**)

The relationships between seasonal ET and yield of tomato for all irrigation treatments applied during both years were well fitted using a linear regression forced to the origin with the regression coefficients (Fig. 2). The slopes of the fitted regressions, which represent the *K*y and the *Kss*, were 0.96 and 0.86 for RDI, while they were 0.87 and 0.77 for the same parameters for CDI, respectively, indicating that in both cases the reduction in crop yield is proportionally less than the relative ET reduction. In this regard, tomato seems to be less sensitive to water deficit in terms of total dry biomass, as the last was less affected by water deficit than fruit yield production. However, the calculation of *K*y for each specific stage of crop growth may help in defending the most critical period of the crop to water.

## Discussion

Tomato plants are one of the most important vegetable crops that are widely grown around the world, but they are also one of the most water-intensive crops [27, 28]. However, deficit irrigation provides a water-saving strategy that has become very popular in arid and semi-arid regions as an important means of reducing water consumption. In 2023, full irrigation resulted in the highest fruit yield and TDM, but there were significant reductions in both parameters when applying less amounts of water. The reductions were less evident between the full irrigation and V80 than between the other deficit irrigation treatments. Several studies report that deficit irrigation typically reduces tomato yield, but the magnitude of the reduction depends strongly on the severity of the water deficit [21, 23, 29]. However, deficit irrigation often lowers total and marketable tomato yield relative to full irrigation, yet the yield penalty is proportional to how severe the water deficit is mild-to-moderate deficits frequently give modest or negligible yield losses, whereas severe deficits cause large declines [29]. In 2024, fruit yield and TDM were significantly depressed with extended reduction of irrigation water during the whole growing season in V50 with respect to full irrigation, since many small green fruits aborted or did not enlarge enough under drier conditions. However, it is important to avoid prolonged stress, which may stunt plant growth and reduce overall biomass. Deficit irrigation, which included deficit of water supply early in the season (V50-100), resulted in better yield and TDM than deficit at the later stage of the growth (V100-50). However, CDI early in the season typically targets the vegetative growth phase, where tomato plants are less sensitive to water stress and could preserve water without severely impacting fruit yield. This result was probably due to the reduction of irrigation water at the early stage, which did not cause enough water stress to affect the formation of tomato yield since the early soil water deficit at the vegetative stage is too early to affect physiological status [1, 30–32]. Moreover, moderate water stress during the vegetative stage has been shown to help roots growth, which makes it easier for plants to get to deeper soil moisture reserves [1, 33]. Similarly, a lower fruit weight per plant occurred as soil water tension during fruit development and maturation increased, which resulted in lower fruit size as also reported by Favati et al., Tao et al. and He et al. [20, 34, 35].

Total dry matter distribution and the values of total HI in 2023 showed a continuous decline with increasing levels of water deficit compared to the control, indicating a low ability of the stressed plants to recover after rewarding. In 2024, HI measurements decreased significantly in response to V50 and late water deficit, which was associated with a reduced TDM distribution among fruits and thus a yield reduction. However, in the (V50-100) treatment, HI remained very close to the control, which was related to the favorable growth conditions promoted by initial canopy development, higher water use, and dry fruit biomass. These results indicate that the continuous growth and fruiting in tomato coincide throughout most of the plant life cycle, particularly during the “late growth phase”. This overlap underscores the importance of regular irrigation during these phenological stages, which are the most sensitive periods to water stress [10, 30].

Efficient water distribution through a deficit irrigation regimes has been identified as a potential approach to improve the sustainability of horticultural production in water-scarce regions. Water productivity increased with a water shortage, and its maximum value corresponded to irrigation treatment receiving lower water supply in V60 and V50 in the years 2023 and 2024, respectively. Although V60 produced the highest WP, indicating its suitability for maximizing yield per unit of water, (V40 in 2023 and V50 in 2024) also maintained relatively high WP under substantially reduced water supply, highlighting the strong potential for water savings. These results suggest that the optimal irrigation strategy depends on management objectives: moderate deficit irrigation favors efficiency and yield stability, while severe water scarcity may be justified under extreme water scarcity, where the importance of water conservation outweighs the cost of yield reduction. This result suggests that the crop can still benefit from the water when this last is supplied to fulfill half of its water requirements. However, it is possible to save water and improve water use efficiency in tomato production, but water should still be supplied throughout the entire growing season even at reduced rates to minimize fruit losses in arid and semi-arid regions [21, 36]. Although identical irrigation management during the whole growth period, as this study, did not achieve maximum water saving, it was easy to operate because water supply during various growth periods could be directly determined according to the changes of crop coefficient (*K*c) and meteorological conditions.

The relationship between total fruit yield and crop ET showed significant differences when the latter decreased across the different irrigation regimes (Table 3). Although the savings in water were similar in both treatments, V50-100 increased WP markedly compared to V100-50 and achieved a superior effect on yield production. Many studies have shown that well-timed deficit irrigation can consistently improve the water productivity of tomato crops, causing only minimal to slight yield reductions compared with full irrigation [1, 11, 37]. This finding is in agreement with previous studies on tomatoes cultivated under a wide range of deficit irrigation treatments [18, 23, 38].

The production function (total volume of applied water versus fresh fruit yield) through linear regression analysis and a mathematical function showed a significant determination factor (R^2^ = 0.979 and 0.903) in 2023 and 2024, respectively (Fig. 2). As expected, the relationship between fruit yield and crop ET was linear (a single line represented all four irrigation treatments), as found in many other crops such as potato (Badr et al.) [39], sweet corn (Ertek and Kara) [40], sunflower (Howell et al.) [41] and green bean (Saleh et al.) [42]. Many studies have also shown that tomato yield responded linearly to the amount of applied water [18, 23, 43].

Crop productivity is strictly linked to the water supply to achieve an acceptable yield quantity and quality. However, tomato plants were more tolerant to deficit irrigation than other vegetable crops such as eggplant (Lovelli et al.; Karam et al.) [44, 45] and potato (Hill et al.; Badr et al.) [46, 47]. The equation model of *K*y proposed by Doorenbos and Kassam [48] allowed for the prediction of crop productivity as a response to their water use by means of the equation reported by Stewart et al. [25]. This factor is crop-specific and indicates the ability of the crop to withstand water stress when its value is less than 1, with a little reduction in yield and stability in water productivity. Given that the observed yield reductions were less than the ET reductions applied under the different irrigation regimes, this may indicate the validity of the yield response factor to water (*K*y) as a synthesized criterion for measuring a crop’s ability to tolerate water stress. At a value of *K*y less than 1, tomatoes show good tolerance to water-deficit regimes with only a slight reduction in yield and significant stability in water productivity. However, tomato seemed to be more sensitive to the severity of the deficit irrigation (throughout the whole growing season) as shown in 2023 under RDI, although the difference between *K*y factors going from the V100 treatment to V40 was sensible, but all values were still lower than one even with V0 when the water supply was cut off very early in the season. On the other hand, reduction of irrigation water early in the season in 2024 under CDI typically results in lower *K*y values compared to late-stage, indicating a more resilient response to early-season water stress. This resilience is attributed to the plant’s ability to compensate for early deficits through enhanced root growth and improved physiological adaptations. Conversely, a reduction of water in the late stage might show higher *K*y values due to increased sensitivity of fruit development processes to water shortage (Fig. 2).

Calculating this coefficient as referring to plant TDM rather than yield, the values of *K*ss in both years were markedly lower than *K*y, which indicates a different behavior for the TDM towards exposure to water deficit. The water regime effect on assimilating distribution and on the yield components may explain the difference in this behavior, where the HI change markedly under water deficit treatments indicates that biomass accumulation of tomato is less affected by soil water deficit than fruit yield, and this is because fruit losses increase with increasing soil water deficit. The slope of the fitted regressions represents the yield response factor for yield, and TDM for all treatments confirms the previous results with the same trend (Fig. 2). Patane et al. [11] stated that the yield response factor was 0.76 for marketable yield (Ky) and 0.49 for total dry biomass (Kss), suggesting that in both cases, the decrease in crop productivity is proportionally less than the reduction in relative ET. These findings suggest that while both RDI and CDI can enhance water productivity, they also lead to a reduction in total fruit yield. CDI appears to be more advantageous than RDI due to its lower impact on yield reduction and lower *K*y value. This implies that strategic timing of water deficits can mitigate adverse effects on crop performance.

Considering the specific character of tomato growth, the yield of each harvest was summed up at the end of the cropping season as the total yield, which was used to calibrate the water-yield models. Zhang et al. [49] reported that the period of fruit growth was most responsible for the improvement of tomato yield, which was also confirmed by Yang et al. [50]. When the early-ripening fruits were harvested, the water deficit would not affect them, but the rest of the fruits were still developing and ripening, which explains the lower fruit weight of the crop. However, due to the indeterminate nature of the tomato growth, the overlaps between fruit development and fruit ripening lead to many times of harvests during the fruit ripening stage, which may affect the productivity of the total yield. Given that all the crop growth stages were not equally sensitive to water stress, the response of tomato to drought stress is complex and depends on the intensity and duration of the stress as well as the developmental stage at which the stress occurs. Xu et al. [21] suggested that the period of fruit development until the onset of color change is the key factor in enhancing tomato yield, a conclusion that was similarly affirmed by Chen et al. [51] and Valcárcel et al. [10]. Finally, deficit irrigation (DI) strategies, such as regulated deficit irrigation (RDI) and controlled deficit irrigation (CDI), hold significant potential for optimizing irrigation water allocation throughout the growing season, thereby enhancing production efficiency by balancing tomato yield and water productivity under water limited conditions.

## Conclusions

Processing tomato, a vital crop for the food industry, requires considerable amounts of water during its growing season. An irrigation strategy based on partial restoration of crop water consumption across the entire season (RDI) showed negative effects on tomato yield, which declined steadily with increasing levels of water stress. In contrast, the timing of water deficits plays a decisive role in determining their effects on yield, as not all growth stages exhibit equal sensitivity to water stress. Imposing deficit irrigation prior to flowering, once plants have developed vigorous vegetative growth (V50-100), appears to offer a balanced approach for applying moderate water deficits, allowing acceptable yields while achieving substantial water saving and improved water productivity. However, careful management is required when applying (CDI) during later growth stages (V100-50) to avoid adverse effects on fruit development and final yield. Therefore, properly timed and well-managed deficit irrigation strategies can achieve an optimal balance between water conservation and yield performance, which is particularly important in arid regions where water scarcity and irrigation costs are increasing.

## Supplementary Information

Supplementary Material 1.

## Data Availability

The datasets analyzed during this study are included in this manuscript.
